# Dependence of ventilation image derived from 4D CT on deformable image registration and ventilation algorithms

**DOI:** 10.1120/jacmp.v14i4.4247

**Published:** 2013-07-08

**Authors:** Kujtim Latifi, Kenneth M. Forster, Sarah E. Hoffe, Thomas J. Dilling, Wouter van Elmpt, Andre Dekker, Geoffrey G. Zhang

**Affiliations:** ^1^ Department of Radiation Oncology H. Lee Moffitt Cancer Center Tampa FL USA; ^2^ Department of Radiation Oncology Mitchell Cancer Institute Mobile AL USA; ^3^ Department of Radiation Oncology (MAASTRO) Maastricht University Medical Centre NL‐6229 ET Maastricht The Netherlands

**Keywords:** ventilation, image registration, deformable, 4D CT

## Abstract

Ventilation imaging using 4D CT is a convenient and low‐cost functional imaging methodology which might be of value in radiotherapy treatment planning to spare functional lung volumes. Deformable image registration (DIR) is needed to calculate ventilation imaging from 4D CT. This study investigates the dependence of calculated ventilation on DIR methods and ventilation algorithms. DIR of the normal end expiration and normal end inspiration phases of the 4D CT images was used to correlate the voxels between the two respiratory phases. Three different DIR algorithms, optical flow (OF), diffeomorphic demons (DD), and diffeomorphic morphons (DM) were retrospectively applied to ten esophagus and ten lung cancer cases with 4D CT image sets that encompassed the entire lung volume. The three ventilation extraction methods were used based on either the Jacobian, the change in volume of the voxel, or directly calculated from Hounsfield units. The ventilation calculation algorithms used are the Jacobian, ΔV, and HU method. They were compared using the Dice similarity coefficient (DSC) index and Bland‐Altman plots. Dependence of ventilation images on the DIR was greater for the ΔV and the Jacobian methods than for the HU method. The DSC index for 20% of low‐ventilation volume for ΔV was 0.33±0.03(1SD) between OF and DM, 0.44±0.05 between OF and DD, and 0.51±0.04 between DM and DD. The similarity comparisons for Jacobian were 0.32±0.03,0.44±0.05, and 0.51±0.04, respectively, and for HU they were 0.53±0.03,0.56±0.03, and 0.76±0.04, respectively. Dependence of extracted ventilation on the ventilation algorithm used showed good agreement between the ΔV and Jacobian methods, but differed significantly for the HU method. DSC index for using OF as DIR was 0.86±0.01 between ΔV and Jacobian, 0.28±0.04 between ΔV and HU, and 0.28±0.04 between Jacobian and HU, respectively. When using DM or DD as DIR, similar values were obtained when comparing the different ventilation calculation methods. The similarity values for the 20% high‐ventilation volume were close to those found for the 20% low‐ventilation volume. The results obtained with DSC index were confirmed when using the Bland‐Altman plots for comparing the ventilation images. Our data suggest that ventilation calculated from 4D CT depends on the DIR algorithm employed. Similarities between ΔV and Jacobian are higher than between ΔV and HU, and Jacobian and HU.

PACS number: 87.57.nj

## I. INTRODUCTION

Radiation pneumonitis has traditionally been cited as the main dose limiting factor in radiation therapy for non‐small cell lung cancer (NSCLC).^(1)^ Previous studies evaluating the risks of pulmonary toxicity have typically reported that the two best predictors were the volume of lung receiving at least 20 Gy^2^, ^3^ and, alternatively, the mean radiation dose to normal lung.^4^, ^5^, ^6^, ^7^, ^8^, ^9^, ^10^, ^11^ To help predict radiation toxicity, many researchers have tried to model the effects of radiation by examining how much normal tissue receives a given dose.^(5)^ There has been much work presented on normal tissue complication probability (NTCP) models.^7^, ^8^, ^9^, ^12^, ^13^

Most of the current models for radiation toxicity of the lung are based on a uniformly functioning lung.^5^, ^7^, ^8^, ^9^, ^12^, ^13^ Although most people have redundant pulmonary reserve, it is well known that lung function is not uniform, and there is a wide range of ventilation and perfusion levels throughout the lung.^14^, ^15^, ^16^ In particular, lung cancer patients have been shown to have regions of lung with poor ventilation. Jeraj et al.^(17)^ suggest that imaging of normal tissue function may be useful in reducing normal tissue toxicity.

The current gold standard for imaging ventilation involves the acquisition of single photon emission computed tomography (SPECT) images. The images are acquired after the patient breathes a radioaerosol (${}^{99m}\text{Tc}-\text{DTPA}$) or radioactive gas. The median diameter of the aerosol particles is close to 1.0μm, making them susceptible to deposition in airways.^16^, ^18^ This deposition may cause artificially high ventilation in some regions of the lung. As a result, the aerosol technique is better at identifying regions with low gamma ray emissions and thus low regional ventilation.^16^, ^19^, ^20^, ^21^, ^22^ Other limitations of SPECT compared to 4D CT include its lower spatial resolution, as well as the longer time needed for image acquisition.^16^, ^23^

Guerrero et al.^(24)^ suggested a pulmonary ventilation imaging algorithm that would calculate the ventilation image from a 4D CT image set. Deformable image registration (DIR) provides a point‐to‐point deformation matrix which is applied to determine the deformation from normal end expiration to normal end inspiration. Guerrero's method uses DIR, and quantifies the density change within a particular voxel between the two end points of the respiratory cycle. The corresponding Hounsfield unit (HU) changes are used to calculate the local ventilation. An algorithm presented by Zhang et al.^25^, ^26^ calculates the ventilation from the volume change (ΔV). The ΔV method is a direct geometrical calculation of the volume change. A specific volume change is obtained by applying the DIR transformation to each of the eight vertex positions of a voxel and then calculating the volume of the deformed volume element. Similarly, the algorithm presented by Reinhardt et al.^27^, ^28^, ^29^ derives ventilation by calculating the Jacobian of the deformation field to approximate the change in volume of voxels. Local volume change of the lung is calculated using the Jacobian of the transformation that maps the end expiration phase of 4D CT image to the end inspiration phase.

The effect of the DIR on the ventilation algorithm is unknown, but if ventilation algorithms are robust, then they will be insensitive to the precise DIR used, provided the DIR is accurate. To test this hypothesis, we investigated the dependence of calculated ventilation on the DIR methods and on the ventilation algorithms. This paper compares ventilation images calculated from 4D CT scans using DIR and the three ventilation algorithms (VA). The DIR algorithms used in this study are optical flow (OF),^30^, ^31^, ^32^, ^33^ diffeomorphic morphons (DM),^34^, ^35^ and diffeomorphic demons (DD).^34^, ^36^, ^37^ The algorithms used for calculating ventilation are HU, ΔV, and the Jacobian. A total of nine combinations of methods are used to calculate ventilation images.

## II. MATERIALS AND METHODS

We used three DIR algorithms (OF, DM, and DD) and three ventilation algorithms (HU,ΔV, and Jacobian) to calculate ventilation images. In order to minimize any registration errors, a $3\times 3\times 3\;{\text{mm}}^{3}$ spatial averaging of all the resulting ventilation images was performed to generate the final ventilation image. In this retrospective study, we examined the 4D CT images acquired at the time of simulation, from ten esophageal and ten lung cancer cases. We used the Dice similarity coefficient (DSC) index to quantify the similarities between the images generated with each method and to study the dependence of the ventilation images on the DIR and VA used. A total of 180 ventilation images, nine for each case, were analyzed.

### A. Deformable image registration methods

The OF algorithm is based on two fundamental assumptions: (1) the intensity change with time of a point in an image is minimal, and (2) the nearby points move in the same manner. This is known as the velocity smoothness constraint. It finds the voxel correspondence by computing a displacement field describing the apparent motion represented in the two images by matching the image intensity gradient.^32^, ^33^, ^38^

The DM algorithm is based on matching of edges and lines.^(34)^ The morphon iteratively deforms a moving image into a target image by morphing the moving image. The process can be divided into three parts: estimation of displacement, accumulation of the deformation field, and deformation. Estimation of displacement is based on quadrature phase difference. The accumulation of the deformation field uses the estimate of the displacement to update the deformation field. Finally, the deformation morphs the moving image to the target image according to the accumulated deformation field. These steps are done iteratively as long as the displacement estimates indicate further morphing to be done.

The methodological basis of the DD algorithm is intensity‐matching. The main requirement is that the voxels in the moving image (M) have the minimal intensity change as the corresponding voxels in the target image (T).^36^, ^39^ Demons forces are applied on the moving image until there is an overlap in intensities between the two. The difference in intensity between the two (M ‐ T) determines the applied force and its direction. When the difference between the two is greater than zero, M moves in the direction of $\vec{\Delta }T$; however, when the difference is less than zero, M moves against $\vec{\Delta }T$. The demons stop exerting force when the images overlap completely.

The deformation field produced by the DM and DD algorithms is smoothed by a Gaussian filter and iteratively used to transform the moving image, and register onto the static image. The DD uses a Gaussian regularization, similar to a diffusion, of the displacement field, which yields smoother deformation fields. Regularization is applied to reduce the influence of extreme values in a deformation field.^(40)^

The OF, DM, and DD DIR were validated by various groups using landmarks, phantoms, and other models.^31^, ^34^, ^41^, ^42^, ^43^, ^44^, ^45^ We also previously validated all three DIR methods using the dataset from a point‐validated pixel‐based breathing thorax model (POPI model), which is a landmark‐based model used for validation of registration algorithms.^(46)^ The methods had a maximum registration error of less than 4 mm or two voxels with insignificant differences between them (p=0.373).^(47)^

### B. Jacobian ventilation

The Jacobian method is a mathematical representation of volume change that uses the first derivative of the deformation field to approximate the change in volume of the voxels.^27^, ^28^, ^29^, ^48^ Local volume change of the lung is calculated using the Jacobian of the transformation that maps the end expiration phase of 4D CT image to the end inspiration phase. Consider a function that represents a vector displacement D(x,y,z) that transforms a voxel from its end expiration image to its corresponding location in the end inspiration image, so that the voxel at (x,y,z) in the end expiration image is displaced by a vector D(x,y,z) to map it to its corresponding location in the end inspiration image. The Jacobian J of this transformation is:

(1) $$J=\text{det}\left[I+\left(\begin{array}{lll} \frac{\partial D_{x}(x,y,z)}{\partial x} & \frac{\partial D_{x}(x,y,z)}{\partial y} & \frac{\partial D_{x}(x,y,z)}{\partial z}\\ \frac{\partial D_{y}(x,y,z)}{\partial x} & \frac{\partial D_{y}(x,y,z)}{\partial y} & \frac{\partial D_{y}(x,y,z)}{\partial z}\\ \frac{\partial D_{z}(x,y,z)}{\partial x} & \frac{\partial D_{z}(x,y,z)}{\partial y} & \frac{\partial D_{z}(x,y,z)}{\partial z} \end{array}\right)\right],$$

where *I* is the identity matrix, $D_{x}(x,y,z)$ is the x component, $D_{y}(x,y,z)$ is the y component, and $D_{z}(x,y,z)$ is the z component of D(x,y,z). The Jacobian operator is used to extract volume changes on a voxel level directly from the deformation field. The determinant of the Jacobian is calculated at each voxel position according to Eq. (1). If the determinant of the Jacobian is zero, then there is no local tissue expansion or contraction; if the determinant is greater than zero, then there is local tissue expansion; and if the determinant is less than zero, then there is local tissue contraction.

### C. ΔV ventilation

The ΔV method is a direct geometrical calculation of the volume change.^(26)^ Each cuboid volume in a CT is composed by eight neighboring voxels as vertices. This cuboid can be used to represent the volume of the voxel. The vertices of the cuboid are changed to create a 12‐face polyhedron. The polyhedron is still comprised by the eight vertices; however, it is now deformed and the correspondence between the deformed vertices and the original ones is established by DIR. Furthermore, the cuboid and the polyhedron are comprised of six tetrahedrons. The volumes of the cuboid and the deformed polyhedron are the sums of the volumes of their corresponding tetrahedrons. During the local volume change calculation, the volume of each voxel is calculated using the corresponding vertices of each respective polyhedron.

The fundamental volume calculation derives from calculating the volume of each tetrahedron. The volume of each tetrahedron is calculated by using the coordinates of its four vertices:

(2) $$V=(\vec{b}-\vec{a})\cdot [(\vec{c}-\vec{a})\times (\vec{d}-\vec{a})]/6,$$

where $\vec{a},\vec{b},\vec{c},\vec{d}$ are the vertices of the tetrahedron as vectors. The volume of a given polyhedron is calculated by summing the volumes of the six tetrahedrons. The coordinates of the deformed tetrahedron are given by the deformation matrix, which is derived from the DIR of the original voxel.

### D. HU ventilation

The HU method uses DIR to correlate voxels from the expiration image set to the anatomically corresponding voxels in the inspiration image. The change in density is calculated by direct comparison of Hounsfield units (HUs).^49^, ^50^ The volume change in the lung due to respiration is because of air volume difference. Therefore, in ventilation calculations using the HU method, the air volume change is calculated using the density change, or HU difference. In a lung voxel in a CT image, the fraction of air is calculated as:

(3) $$F_{air}=-\frac{HU}{1000}$$

Let $F_{\text{exh}}$ be the fraction of air in a voxel in the exhale CT volume, and $F_{\text{in}}$ the fraction of air in the corresponding voxel in the inhale CT volume, then the volume change in the voxel is:

(4) $$\frac{\Delta V}{V_{ex}}=\frac{F_{in}-F_{exh}}{F_{exh}(1-F_{in})}$$

By substituting Eq. (3) into Eq. (4), we get:

(5) $$\frac{\Delta V}{V_{ex}}=\frac{-\frac{HU_{in}}{1000}+\frac{HU_{exh}}{1000}}{-\frac{HU_{exh}}{1000}(1+\frac{HU_{in}}{1000})}$$

Simplifying further, we get to the final equation that relates volume change to density change in the corresponding voxel:

(6) $$\frac{\Delta V}{V_{ex}}=1000\frac{\left(HU_{in}-HU_{exh}\right)}{HU_{exh}(1000+HU_{in})}$$

### E. Dice similarity coefficient (DSC) and Bland‐Altman plots

DSC index is a measure of the degree of overlap between two areas or volumes.^51^, ^52^ When comparing a reference volume A to another reference volume B, the Dice similarity coefficient is:

(7) $$DSC(A,B)=\frac{2\times \left|A\cap B\right|}{\left|A\right|+\left|B\right|}$$

The values of DSC index range between 1.0 and 0.0. A DSC index of 1.0 indicates a complete overlap of the two methods examined, whereas a DSC index of 0.0 indicates no overlap between the methods examined, and intermediate values describe proportional amount of overlap.

Dice similarity coefficient analysis was performed on the ventilation images. The region of lower 20% ventilation threshold in one image was compared to the lower 20% ventilation in the second image. The overlap or the similarity between the two volumes was calculated using the DSC index. Additionally, the volumes describing the regions with an upper 20% ventilation threshold were compared using the DSC index. The volume with lower 20% ventilation includes all the voxels that have ventilation values below 20% threshold in the entire lung image. Similarly, the volume with upper 20% ventilation includes all the voxels that have values above 80% in the entire lung image.

**Figure 1:**
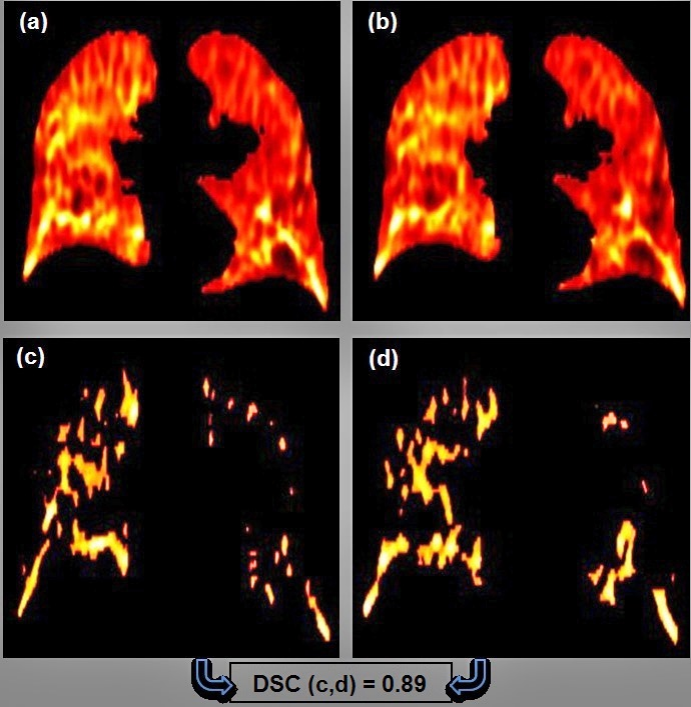
Illustration of the upper 20% thresholded ventilation: (a) and (b) represent the derived ventilation with ΔV and Jacobian algorithms (optical flow was used for DIR in this case); (c) and (d) represent the volumes of the upper 20% threshold of the ventilation in (a) and (b), respectively.

Figure 1 is an illustration of images thresholded for calculating the DSC index. The figure shows the upper 20% ventilation; the lower 20% thresholding was done in a similar manner. Figures 1(a) and (b) show ventilation calculated with ΔV and Jacobian algorithms, respectively, OF was used for DIR. Figure 1(c) shows the thresholded regions of Fig. 1(a), and Fig. 1(d) shows the thresholded regions of Fig. 1(b).

Additionally, Bland‐Altman plot analysis was performed on the ventilation images.^(53)^ The Bland‐Altman method creates scatter plots. The differences between two measurements are plotted on the y‐axis, and the average of the two measurements is plotted on the x‐axis. Generally, if the average of the differences between the two methods is close to zero, this indicates that the two methods produce similar results. The 95% confidence limits are shown as two dotted lines. The closer these limits of agreement are to zero, or to the average of the differences, the more similar the two measurements are.

### F. Ventilation dependence on the DIR and ventilation algorithm

We compared ventilation images calculated with the three DIR methods by calculating the DSC index between images that were calculated with the same ventilation algorithm but different DIR method. The process was repeated for images calculated with the second, and then the third ventilation algorithm.

To calculate ventilation dependence on the ventilation algorithm, images calculated with the three different VAs but same DIR, were compared to each other via the DSC index, then the process was repeated for images calculated with the second and third DIR method.

4D CT sets from 20 patients, ten lung and ten esophageal cancer patients, treated with external beam radiotherapy were selected for the retrospective study. 4D CT image sets were collected on a Philips Large Bore Brilliance 16 slice scanner (Philips Oncology Systems, Cleveland Ohio). The CT sinogram data were binned into 10 phases based on bellows on the abdomen using the method described by Keall et al.^(54)^ The pixel size in the transaxial slice of the 4D CT images for 13 patients was $1.17\times 1.17\;{\text{mm}}^{2}$. The slices for seven patients had a pixel size of $0.97\times 0.97\;{\text{mm}}^{2}$, and the slice thickness was 3 mm.

## III. RESULTS

### A. Ventilation dependence on the DIR

**Figure 2:**
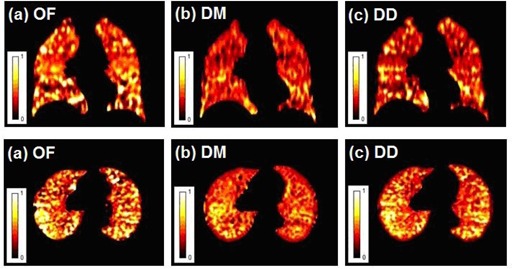
Coronal and axial slices of the ΔV ventilation images for a representative patient with OF (a), DM (b), and DD (c) deformation.

Figures 2(a), (b), and (c) show coronal and axial images of the calculated ventilation using the ΔV ventilation method with the OF, DM, and DD DIR algorithms. Bright colors in the images show high ventilation and dark colors show low‐ventilation regions. Some of the high‐ and low‐ventilation areas seem to correspond between the different methods; however, the overall images are quite different from each other.

**Figure 3:**
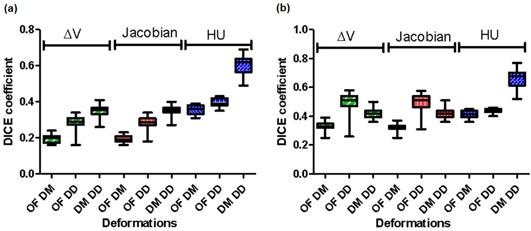
Dice similarity coefficient (DSC): comparisons (a) between OF, DM, and DD deformation with ΔV, Jacobian, and HU ventilation for the lowest 20% ventilation, and (b) for the highest 20% ventilation.

Figures 3(a) and (b) are plots of the DSC index for the lowest and highest 20% ventilation for all 20 patients, respectively, for the ΔV, Jacobian, and HU methods calculated using the OF, DM, and DD DIR algorithms. Figure 3(a) shows the DSC index in the lower ventilation range (0%–20%) for all 20 patients comparing the ΔV, Jacobian, and HU ventilation. The DSC index for 20% of low‐ventilation volume for ΔV was 0.33±0.03 between OF and DM, 0.44±0.05 between OF and DD, and 0.51±0.04 between DM and DD. The similarity comparisons for Jacobian were 0.32±0.03,0.44±0.05, and 0.51±0.04, respectively, and for HU they were 0.53±0.03,0.56±0.03, and 0.76±0.04, respectively. The DSC index for the upper ventilation (80%‐100%) is plotted in Fig. 3(b) and showed trends similar to the lower ventilation.

### B. Ventilation dependence on the ventilation algorithm

**Figure 4:**
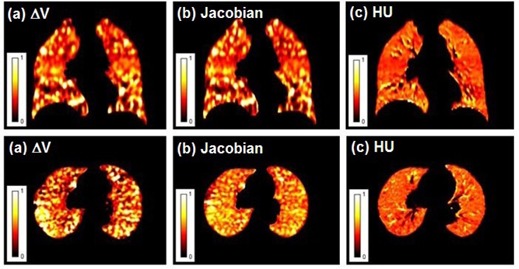
ΔV (a), Jacobian (b), and HU (c) ventilation images for a representative patient with OF deformation.

Figures 4(a), (b), and (c) show coronal and axial images of the calculated ventilation using the ΔV, Jacobian, and HU ventilation methods with the OF DIR algorithm. By visual observation of Fig. 4, we can see that there is a higher degree of overlap between the ΔV and the Jacobian ventilation images.

**Figure 5:**
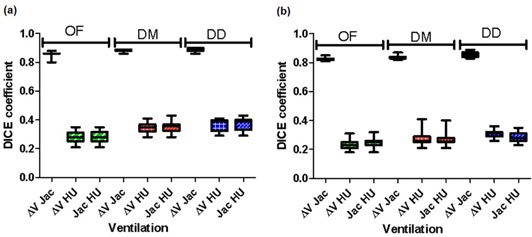
DSC index (a) for OF, DM, and DD deformation for the lowest 20% ventilation for comparison between ventilation algorithms; DSC index (b) for OF, DM, and DD deformation for the highest 20% ventilation for comparison between ventilation algorithms.

The DSC index for using OF as DIR, as shown in Fig. 5, was 0.86±0.01 between ΔV and Jacobian, 0.28±0.04 between ΔV and HU, and 0.28±0.04 between Jacobian and HU, respectively. The DSC index for using DM as DIR was 0.88±0.01 between ΔV and Jacobian, 0.34±0.04 between ΔV and HU, and 0.35±0.04 between Jacobian and HU, respectively, and for DD it was 0.88±0.01,0.36±0.04, and 0.37±0.04, respectively. The DSC index values for the highest 20% ventilation were similar to the ones for the lowest 20% ventilation.

**Figure 6:**
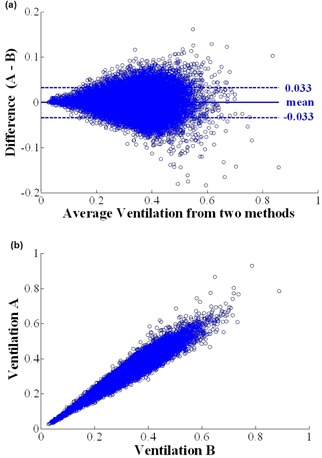
Voxel‐to‐voxel comparison of two ventilation images from a representative patient calculated using the Jacobian and ΔV ventilation algorithm and DM as DIR: (a) Bland‐Altman plot comparing the Jacobian and ΔV ventilation, and (b) scatter plot comparing the two methods.

In addition to the DSC index, Bland‐Altman plots were utilized to study the ventilation maps. Figure 6(a) shows the Bland‐Altman plot for a representative patient. This case compared the ΔV and Jacobian ventilation with DM as the DIR. The mean of the differences was zero and the range of 95% confidence limits was −0.03 to +0.03. Figure 6(b) is a scatter plot of the Jacobian and ΔV ventilations with DM as DIR; correlation coefficient for this case was 0.96. Table 1 shows the 95% confidence limits resulting from the Bland‐Altman plots, along with the correlation coefficient (CC) between the algorithms.

Table 2 shows a summary of the 95% confidence interval derived from the Bland‐Altman plot, as well as the correlation coefficient between the ventilation images. Similar to the DSC index, it shows that the ΔV and Jacobian have the highest correlation between them.

**Table 1:** Bland‐Altman statistics for comparisons between OF, DM, and DD deformation with ΔV, Jacobian, and HU ventilation

*Vent*.	ΔV			*Jacobian*			*HU*		
DIR	OF	OF	DM	OF	OF	DM	OF	OF	DM
	DM	DD	DD	DM	DD	DD	DM	DD	DD
95%	±0.33	±0.28	±0.16	±0.33	±0.28	±0.16	±0.46	±0.32	±0.26
CC	0.21	0.52	0.50	0.20	0.52	0.50	0.26	0.32	0.66

**Table 2:** Bland‐Altman statistics for comparisons between ventilation algorithms showing the 95% confidence limits and the correlation coefficient between the methods

*DIR*	*OF*			*DM*			*DD*		
DIR	ΔV&J	ΔV&HU	J&HU	ΔV&J	ΔV&HU	J&HU	ΔV&J	ΔV&HU	J&HU
95%	±0.12	±0.45	±0.43	±0.03	±0.49	±0.49	±0.05	±0.26	±0.26
CC	0.93	0.11	0.13	0.96	0.13	0.13	0.96	0.31	0.30

## IV. DISCUSSION

In agreement with Castillo et al.,^(48)^ our results show that the ΔV and the Jacobian ventilation calculation methods are very similar. Both qualitative and quantitative inspections show that these two have a high degree of similarity when using the same DIR method. DSC index between the ΔV and Jacobian is near 0.9, indicating nearly 90% overlap between the ventilation images calculated with the two methods. The difference between the two methods is their mathematical implementation. While the Jacobian estimates ventilation by calculating the first derivative of the deformation field, the ΔV estimates ventilation by direct geometrical calculation of the volume change. Bland‐Altman plots also confirm the results of the DSC index when comparing the ventilation methods. According to these plots, the ΔV and Jacobian provide nearly identical ventilation results with a correlation coefficient of 0.96 when using DM as DIR, with a mean of differences at zero, and the confidence interval at ± 0.03.

When compared to the HU method, ΔV and Jacobian are very different. DSC index shows only about 30% similarity between these methods, and the HU method (see Fig. 5). The HU method is a density‐based ventilation calculation method. The DSC index, shown in Fig. 3, suggests that the HU method is less dependent on the DIR used. It depends more on the CT image quality due to the inherent noise of HUs in normal CT imaging. The fluctuations of the HUs in a CT lead to noisy ventilation images. The standard deviation of HU in a CT image of a water phantom similar to the change in HU between inspiration and expiration.^(26)^

If most of the lung is similar in density (i.e., a mixture of blood vessels and alveoli), then the effects of any misregistrations will be small because of the similar densities. However, if there is a misregistration between lung and much denser tissue, such as chest wall or large artery, then the change in HU is quite significant. The HU algorithm screens for these voxels with very large change in HUs and then excludes these voxels in the ventilation image. To overcome the variation in HUs in low‐density tissue, Castillo et al.^(48)^ smoothed the DIR using a cube of 5×5×5 voxels, then also smoothed the ventilation image using a box filter 9×9×3 voxels. In contrast, in our study no additional smoothing was applied to the DIR, only the ventilation images were smoothed with a $3\times 3\times 3\;{\text{mm}}^{3}$ box filter.

The three DIR algorithms used in this study produced similar results when tested for accuracy using landmarks. The fact that the ventilation maps are different suggests that the dependence of ventilation calculation on DIR algorithms is fundamentally due to the discrepancies between the vector fields generated by different DIR algorithms. Figure 3 suggest that there is a higher similarity between DM and DD than between OF and DM, or OF and DD. This may be due to the different vector fields that are produced from these methods. The similarity between DM and DD may be a result of both methods using similar filters to smooth their deformation fields and the fact that both of them are diffeomorphic, whereas this does not apply to the OF algorithm. The difference between these three DIR algorithms and their vector fields will be further investigated in a future study. Furthermore, the sliding motion near lung boundary could lead to additional artifacts in the DIR, which would lead to false ventilation results. This issue was not taken into account in this study and will be considered for future projects.

The need for smoothing for the ΔV and Jacobian methods comes from these misregistrations of the end expiration and inspiration image sets. The source of the misregistration issues comes directly from the 4D CT itself. Currently there are two commercially available techniques for 4D CT imaging. The first uses a cine acquisition (GE Medical Systems, San Francisco, CA), which rapidly bins axial images based on a breathing trace. This method produces slab artifacts that are clearly visualized on sagittal and coronal reconstructions with the width of each slab corresponding to the transaxial collimation used in the 4D CT acquisition. The second acquisition technique bins the sinogram data, then reconstructs each phase on these binned sinogram data (Philips, Philips Healthcare, Andover, MA; and Siemens, Siemens Medical Solutions, Malvern, PA). The artifacts are generally less pronounced in this imaging technique, though both methods produce significant artifacts when patients breathe irregularly. Due to limitations of CT scanners and the ability of a patient to breathe reproducibly, most 4D image sets present with imaging artifacts that are unique to 4D acquisitions.^(55)^

In general, a higher DSC value means a better similarity between the images. But we cannot determine what value of DSC is acceptable from this study. In the study by Castillo et al.,^(48)^ ventilation calculated from 4D CT was compared to that measured with SPECT for the HU and volume change ventilation algorithms. The Castillo study results showed that the calculated ventilation had a good agreement with that measured using SPECT; however, in that study only one DIR algorithm was used. In the future, to establish a better understanding of the DSC values obtained from our study, the ventilation calculated from 4D CT using these ventilation methods and various DIR algorithms needs to be compared to ventilation from SPECT, which is widely considered gold standard in ventilation imaging.

## V. CONCLUSIONS

DSC index analysis suggests that ventilation calculated from 4D CT depends on the DIR algorithm employed. We believe that artifacts in 4D CT images are the reason why HU shows a smaller dependence on the choice of DIR. When comparing ventilation algorithms with each other, we found that similarities between ΔV and Jacobian are higher than between ΔV and HU and between Jacobian and HU. This shows that ΔV and Jacobian are similar, while HU is a different ventilation calculation method (inferring volume changes from changes in HUs) and is more sensitive to noise in HUs.

## ACKNOWLEDGMENTS

This work was partially supported by a grant from the Varian Medical Systems, Inc.
